# Factors Influencing the Health-Related Quality of Life of Workers According to the Type of Work

**DOI:** 10.3390/healthcare10102066

**Published:** 2022-10-18

**Authors:** Sunae Kim, Myoungjin Kwon, Kawoun Seo

**Affiliations:** 1 Department of Nursing, Korea National University of Transportation, Cheongju 27909, Korea; 2 Department of Nursing, Daejeon University, Daejeon 34520, Korea; 3 Department of Nursing, Joongbu University, Geumsan 32713, Korea

**Keywords:** daytime workers, quality of life, regression, shift workers, survey

## Abstract

This paper describes a descriptive cross-sectional study that was conducted to identify and compare the factors affecting health-related quality of life (HRQoL) according to the type of work. The method involved a secondary analysis of 4131 workers who participated in the first year of the 8th Korea National Health and Nutrition Examination Survey (2019). In this study, a complex sample plan file was created and then weighted and analyzed. For the analyses, frequency, χ^2^-test, *t*-test, and linear regression analyses were used for complex sample analysis. Factors that significantly affected the HRQoL of daytime workers were educational background, living with a spouse, regular work, depression for two consecutive weeks, and suicidal thoughts. The explanatory power was 18.9% (*p* < 0.001). The factor that significantly affected the HRQoL of shift workers was whether they took dietary supplements for 2 weeks or more during the past year, and the explanatory power was 17.6% (*p* = 0.007). This study confirmed that the factors affecting HRQoL differ according to the type of work. Based on the results of this study, when developing a program to improve the HRQoL of workers, it is necessary to establish a differentiated strategy according to the type of work.

## 1. Introduction

Quality of life (QoL) is a multidimensional concept in which self-awareness of an individual’s current psychological state is important [1]. Among its various domains, health-related QoL (HRQoL) is a concept with a special focus [2]. The type of work is the largest framework for classifying working hours and is generally divided into daytime work and shift work. Daytime work involves going to work between 7:00 a.m. and 9:00 a.m. and leaving the workplace between 4:00 p.m. and 6:00 p.m. [3]. Shift workers report more insomnia, sleep disturbance, and fatigue than daytime workers due to irregular working hours [4]. It was also reported that shift workers are more stressed than nonshift workers and have lower QoL because of mental health issues such as obsessive–compulsive disorder, sensitivity, depression, anxiety, and hostility regarding these issues [5]. Because these problems can also be linked to workplace accidents in certain occupations, efforts have been made to examine and manage the HRQoL of shift workers [6].

However, with the recent developments in science and technology and the emergence of efficient work methods, there has been a diversification of work types [7]. Shift work, which has been implemented in some occupations such as nursing and policework and requires continuity of service for 24 h, has been taking various forms due to social changes and demands. Shift work has become essential for a 24 h society [8]. In the United States, 20–25% of all workers are engaged in various types of shift work [9], while in Korea, 15.2% of workers work on a shift basis [10]. In addition, because daytime work has also taken various forms, the QoL related to the health of workers may not be limited only to shift workers. Additionally, stemming from the recent introduction of the flexible working system, there is a need to compare the factors affecting the QoL according to type of work, which can vary according to the individual’s propensity [7].

Furthermore, health and illness status have been emphasized as factors affecting HRQoL [11]. In previous studies, depression, average monthly income, and spousal relationship were reported as factors influencing the QoL of regular workers [12]. For female workers, working conditions, job stress, education level, and disease have been found to be influencing factors [13,14]. The factors influencing the QoL of wage earners were age, economic level, family size, spouse cohabitation, depression, and subjective health [15]. For the self-employed, these were age, number of lunches per week, drinking frequency, number of days of strength training, body mass index (BMI), stress, and subjective body type recognition and health [15]. In addition, smoking and drinking, which are important health behaviors, were also factors affecting the QoL [16]. The variables judged to have an impact on the quality of life of workers through previous studies were subjective health status and subjective body type perception. In relation to health status, obesity, aerobic physical activity, drinking, smoking, and sedentary time have also been found to be influential [12,14,15]. Research on the HRQoL of workers has included studies on regular and nonregular workers [12], wage earners and self-employed people [15], and factors affecting quality of life by gender [14]. Although many studies have been conducted on factors affecting the QoL of workers, there are only a few cases in which these have been identified by distinguishing between daytime workers and shift workers.

Therefore, this study aimed to identify factors affecting the QoL of workers according to the characteristics of their work by classifying shift work as all-day, evening, and night work. The data used in this study were from the Korea National Health and Nutrition Examination Survey (KNHANES), which is representative of the entire Korean population, and its reliability has been verified. Hence, the results of the analyses are significant and have the advantage of being generalizable for all Korean workers. It is also meaningful to utilize the results measured through the Health-Related Quality of Life Instrument with 8 Items (HINT-8), which has been used as a measuring tool for HRQoL since 2019. HINT-8 has the advantage of being able to express QoL in detail because there are more domains or health states where it can express health status than Euro QoL–5 Dimension (EQ-5D), which is another existing HRQoL measurement tool. In addition, this study attempted to provide basic data for establishing strategies tailored to each type of work, to mediate the diversity of work types and problems that inevitably arise in the 24 h working environment.

## 2. Materials and Methods

### 2.1. Study Design and Participants

This study is a secondary analysis of data from the first year of the 8th KNHANES 2019. It is a descriptive research study aimed at understanding the factors affecting the HRQoL of workers according to the type of work in which one is engaged.

The KNHANES calculates representative and reliable statistics at the national and provincial level to understand the public health status, health behavior, and food and nutrition intake and is conducted by the Ministry of Health and Welfare and the Korea Centers for Disease Control and Prevention. This is a nationwide health and nutrition survey to be used as basic data for health policies, such as setting goals and evaluation plans, and developing health promotion programs. Participants of the survey filled out a consent form. In this study, 4131 workers out of 8110 people in the first year of the 8th KNHANES were targeted. Participants who clearly responded regarding their work type were selected. Among them were 3425 people who said they worked from 9 a.m. to 6 p.m. and 706 who worked in shifts. Shift work refers to continuing work at a different time from that of day work, such as evening work (14:00~21:00), night work (21:00~8:00 next day), regular day/night shift work, or 24 h shift work [17].

### 2.2. Study Variables

Demographic factors studied included sex, age, income level (upper, middle, and lower), education level (middle school or lower, high school, and college or higher), living with a spouse, taking dietary supplements (vitamins, minerals, etc.) for more than 2 weeks in the past year, and HRQoL. HRQoL was measured using HINT-8. HINT-8 is a tool developed using qualitative and quantitative research methods to measure the HRQoL of Koreans, and consists of 8 items in 4 health areas (physical, social, mental, and positive). On a 4-point scale, 1 indicates no problem, and 4 indicates serious problems, with lower values indicating higher HRQoL [18]. Labor-related factors were occupation (manager, service worker, or craftsman) and occupational status (wage worker or self-employed). Occupations were classified into manager (manager, expert, office worker), service worker (service worker, sales worker), operator (technical worker, machine operator), and craftsman (agricultural, forestry, fishery worker, simple labor worker). With regard to occupational status, the self-employed were distinguished as employers or unpaid family workers.

Physical factors considered included high blood pressure, diabetes, obesity, aerobic physical activity, alcohol consumption, smoking, and time spent sitting per day. Hypertension and diabetes were established through a diagnosis by a doctor. Obesity was defined as underweight (BMI less than 18.5 kg/m^2^), normal (BMI less than 18.5 to 25 kg/m^2^), and obese (BMI greater than or equal to 25 kg/m^2^) [19]. Aerobic exercise practice referred to whether the individual practiced moderate-intensity physical activity for 2 h 30 min or more, or high-intensity physical activity for 1 h 15 min or more, or a mix of moderate- and high-intensity physical activity for the equivalent time period each week (1 min of high intensity is considered equivalent to 2 min of moderate intensity) [20]. Emotional factors included subjective health status (good, average, or bad), subjective body type recognition (thin, average, or obese), stress (feeling less or feeling more), feeling depressed for 2 consecutive weeks, and experiencing thoughts of suicide.

### 2.3. Statistical Analyses

In this study, 4131 workers’ data were extracted from the raw data of the 2019 KNHANES, collected by the stratified colony extraction method, to identify factors affecting HRQoL. After creating a complex sample plan file using the IBM SPSS 25.0 program (IBM Corp., Armonk, NY, USA), weights were assigned and analyzed, and the significance level was set at 0.05. The degree of each sociodemographic, physical, and psychological factor was analyzed using frequency analysis among the complex sample analysis. For frequency, actual values were used, and for percentages, numerical values considering weights were used. The differences in demographic factors, physical factors, and psychological factors according to work type were analyzed by cross-analysis and t-test during the complex sample analysis. For factors affecting HRQoL, linear regression analysis was used in the complex sample analysis.

## 3. Results

### 3.1. Comparison of Sociodemographic Factors

There were differences between groups in terms of age, income level, education level, cohabitation with a spouse, whether they took dietary supplements for two weeks or more in the past year, occupation, and smoking status (Table 1). Age was higher among daytime workers (t = 4.70, *p* < 0.001), and the income level was higher for shift workers (χ^2^ = 2.20, *p* = 0.001). More daytime workers were found to have a higher level of education compared with shift workers (χ^2^ = 53.97, *p* < 0.001), and the number of daytime workers living with their spouses was also higher compared with shift workers (χ^2^ = 7.36, *p* = 0.012). In the previous year, more daytime workers were consuming dietary supplements compared with shift workers (χ^2^ = 5.58, *p* = 0.033), managers were mostly daytime workers, and service workers were mostly shift workers (χ^2^ = 140.11, *p* < 0.001). Smokers were more common among shift workers (χ^2^ = 6.02, *p* = 0.047).

### 3.2. Comparison of Physical and Psychological Factors

There were differences between the groups in terms of whether or not the individuals practiced aerobic physical activity, felt depressed for two consecutive weeks, had suicidal thoughts, and HRQoL (Table 2). Shift workers were more involved in aerobic physical activity (χ^2^ = 8.58, *p* = 0.017). Additionally, they reported more depression over two consecutive weeks (χ^2^ = 9.52, *p* = 0.005) and suicidal ideation than the daytime workers (χ^2^ = 18.82, *p* < 0.001). HRQoL was higher among shift workers (t = −2.99, *p* = 0.003).

### 3.3. Factors Affecting HRQoL

In order to identify the factors affecting HRQoL, linear regression analysis was performed by inputting factors that showed a significant difference in the crossover analysis and t-test as independent variables. The regression equation for HRQoL of daytime workers was found to be significant (F = 7.29, *p* < 0.001), and the explanatory power was 18.9% (Table 3). Factors that had a significant influence were middle school graduate (B = 1.486, *p* = 0.005), living with a spouse (B = −1.253, *p* = 0.030), and regular workers (B = −0.759, *p* = 0.016), no depression (B = 3.044, *p* < 0.001), and no suicidal ideation (B = 2.921, *p* < 0.001).

The regression equation for HRQoL of shift workers was found to be significant (F = 2.31, *p* < 0.001), and the explanatory power was 17.6%. The factor that had a significant effect was the intake of dietary supplements (B = 1.262, *p* = 0.032).

## 4. Discussion

This study was conducted to investigate the factors affecting the HRQoL according to the working patterns of daytime workers and shift workers using the KNHANES data. The findings of the study indicated that the HRQoL was higher among shift workers than daytime workers, and the income level of shift workers was also higher. This is consistent with the results of a previous study on nurses [21], which observed that the high-income ratio of shift workers was higher than that of daytime workers. In general, in the case of shift workers, it can be expected that their income is higher owing to the shift allowances being included in the salary. However, in another study on women workers, there was no difference in income between daytime and nighttime workers, which is different from the results of this study [22]. This could be because salary depends not only on the type of work but also on the position and job performance. In fact, in this study, the ratio of daytime work was high among managers.

This study found that the HRQoL of shift workers was higher than that of daytime workers. There was a difference in the results compared with the previous studies in that the QoL of university workers was lower [23]. However, in a previous study targeting Croatian hospital nurses, it was reported that the HRQoL of shift workers was not significantly lower than that of daytime workers, which is similar to the results of this study [24]. This may be because the characteristics of the occupations were not reflected in this study. Therefore, further research is needed to compare the factors affecting HRQoL by occupation. 

In this study, in the case of dietary supplements, daytime workers consumed more, but previous studies showed that shift workers consumed more nutritional supplements than daytime workers. However, it is difficult to simply compare the current study with the prior study as its data only covered male workers employed in national industrial complexes. Considering that dietary supplements intake was high among the middle and older age groups, the average age of daytime workers was 45, and the average age of shift workers was 41, it can be interpreted as a result reflecting the age difference. Although shift work may negatively affect health, the fact that shift workers consume fewer dietary supplements than daytime workers does not mean that they are less concerned about their health. However, there is a need to conduct further studies on their health-related behaviors.

As a result of this study, it was found that shift workers smoked more than daytime workers and had unhealthy behaviors. However, there was no significant difference in smoking between the two groups. This result is consistent with those of previous studies reporting that shift-working men and women smoke more than day workers [25]. Therefore, shift workers should be recognized as a high-risk group for smoking, which should be considered a priority in health education.

In this study, aerobic physical activity was also found to be higher in shift workers than in daytime workers. In a previous study, 65.6% of shift workers responded that they exercised more than 3 days a week and 30 min or more a day, whereas only 44.9% of full-time workers responded that they exercised. This result was significant and consistent with the results of this study [26]. This may be because shift workers are aware that there are many health risk factors caused by irregular lifestyles, and exercise is a part of an effort to improve health-related outcomes. However, there was no significant difference in smoking between the two groups in the aforementioned study. It is inferred that the behavioral practice rate is low, which is in partial agreement with the results of this study. 

The rates of depression and suicidal thoughts were significantly higher for shift workers, confirming that there were associated risk factors for their mental health as well. These results are consistent with those of previous studies showing that shift workers are more likely to have mental health problems [5]. Therefore, it is suggested that interventions to improve the mental health of shift workers are needed in the future.

A finding of this study is that the lower the education level of the daytime workers, the higher the HRQoL. Additionally, the participants without depression or suicidal thoughts had higher HRQoL. The intake of dietary supplements was confirmed as a factor affecting the HRQoL of shift workers, and it was higher for workers who took them. In general, the HRQoL of workers is reported to be lower as mental stress such as depression increases, which is consistent with the results of this study [27]. However, as the previous research was conducted by dividing the types of work, a simple comparison is not possible. In addition, it was reported that the higher the social support, including the spouse’s support, the higher the HRQoL [28]. Living with a spouse can be thought of as a form of social support. Therefore, whether they are living with their spouse or only being supported by them, the presence of a spouse is an important factor affecting their QoL. For shift workers, dietary supplement intake was analyzed as the only factor affecting HRQoL. Shift workers may not have regular meal times or may have difficulty eating with others due to irregular schedules. Therefore, it is thought that they may have taken dietary supplements as an effort to not decrease their HRQoL. In particular, because HRQoL involves a strong self-awareness aspect of their health status, it can be inferred that shift workers thought that irregular work patterns would have a detrimental effect on their health, so they chose to consume dietary supplements. Therefore, taking dietary supplements taken by shift workers is considered to be one factor that increases their HRQoL.

In this study, the HRQoL was analyzed using HINT-8, a tool that was first used to measure the factor in the 2019 KNHANES. It has great significance as a new analysis tool. Although this study has the advantage of being one of the first of its kind, it is difficult to simply compare and interpret the results. There is a large difference in the number of participants included in each of the two groups, and there is no previous study that evaluated workers’ HRQoL using HINT-8, so comparisons are difficult. Therefore, it is evident that there are limitations in presenting the results because there are no comparable prior studies. In the process of integrating the levels of each domain, it was reported as a result that the response contents of each level were not properly reflected. However, rather than a simple comparison, it is recommended to compare the levels by using a conversion formula by mapping between tools. In addition, this study is a secondary analysis study and did not include variables such as family-related or living standards that are expected to affect workers’ quality of life. Therefore, in the future, it is necessary to consider factors such as sex and age in more detail and to include variables such as family and living standards. Although the numbers of subjects in the day-worker and shift-worker groups were not small, this study had a large difference in the number of subjects in each of the two groups. Therefore, it is necessary to consider the number of subjects between groups in future studies. In addition, as this study is cross-sectional, it is difficult to derive causal relationships; it is necessary to confirm the causal relationships through a longitudinal study using the longitudinal data for this study variable.

## 5. Conclusions

This was an initial study conducted to measure HRQoL using the HINT-8 tool, and it aimed to identify the factors affecting QoL by classifying work patterns into daytime and shift work. As a result of this study, it was confirmed that the factors affecting the QoL of daytime workers and shift workers were different, and the differences from previous studies were confirmed. However, it is difficult to simply interpret the results in the absence of previous studies using the same tools. Studies that identify factors affecting QoL by classifying working types into daytime and shift work are insufficient. The findings of this study can be used as fundamental data to establish the foundation for such research.

## Figures and Tables

**Table 1:** Comparison of sociodemographic factors (N = 4131).

Characteristics		Daytime Work (*n* = 3425) N (Weight %)/m (SE)	Shift Work (*n* = 706) N (Weight %)/m (SE)	x^2^/t (*p*)
Sex	Male	1741 (56.1)	367 (57.7)	0.65 (0.558)
	Female	1684 (43.9)	339 (42.3)	
Age		45.15 (0.74)	41.42 (0.79)	4.70 (<0.001)
Income level	Upper	707 (20.1)	193 (27.6)	2.20 (0.001)
	Middle	1796 (53.1)	357 (50.7)	
	Lower	916 (26.8)	152 (21.7)	
Education level	≤Middle school	756 (16.7)	138 (14.0)	53.97 (<0.001)
	High school	1103 (32.9)	315 (47.3)	
	≥College	1566 (50.4)	253 (38.7)	
Living with spouse	Yes	2418 (90.4)	396 (86.2)	7.36 (0.012)
	No	340 (9.6)	79 (13.8)	
Intake of dietary supplements	Yes	1777 (59.6)	329 (54.4)	5.58 (0.033)
	No	1164 (40.4)	259 (45.6)	
Occupation	Manager	1325 (48.1)	162 (27.1)	140.11 (<0.001)
	Service workers.	535 (18.0)	218 (37.7)	
	Technicians	1116 (33.8)	231 (35.2)	
Employment status	Wage workers	2262 (77.3)	453 (73.8)	3.50 (0.140)
	Self-employed	732 (22.7)	161 (26.2)	
Regular workers	Yes	1148 (55.1)	140 (36.3)	52.87 (<0.001)
	No	1106 (44.9)	296 (63.7)	
Alcohol drinking	Yes	2072 (63.7)	430 (63.8)	0.01 (0.978)
	No	1347 (36.3)	275 (36.2)	
Smoking	Yes	664 (46.8)	178 (54.0)	6.02 (0.047)
	No	847 (53.2)	160 (46.0)	

**Table 2:** Comparison of physical, psychological factors (N = 4131).

Characteristics		Daytime Work (*n* = 3425) N (Weight %) /m (SE)	Shift Work (*n* = 706) N (Weight %) /m (SE)	x^2^/t (*p*)
Hypertension	Yes	698 (17.4)	134 (15.6)	0.34 (0.898)
	No	2698 (82.6)	548 (84.4)	
Diabetes	Yes	255 (6.4)	60 (6.9)	0.01 (0.928)
	No	3141 (93.6)	622 (93.1)	
Obesity	Underweight	117 (3.7)	22 (4.0)	1.76 (0.557)
	Normal	2079 (60.5)	418 (62.9)	
	Obese	1177 (35.8)	241 (33.2)	
Aerobic physical activity	Yes	1531 (47.0)	340 (53.0)	8.58 (0.017)
	No	1859 (53.0)	341 (47.0)	
	No	1347 (36.3)	275 (36.2)	
	No	847 (53.2)	160 (46.0)	
Sitting time per day		8.83 (0.13)	9.18 (0.37)	−0.93 (0.354)
Subjective health	Healthy	1161 (35.0)	242 (35.7)	5.13 (0.145)
	Moderate	1809 (52.4)	351 (48.9)	
	Unhealthy	455 (12.6)	113 (15.5)	
Subjective body image	Thin	487 (14.5)	104 (17.0)	4.78 (0.198)
	Moderate	1417 (40.3)	283 (41.6)	
	Obese	1516 (45.2)	318 (41.4)	
Stress	Feel less	2450 (69.7)	497 (69.3)	0.04 (0.857)
	Feeling a lot	969 (30.3)	208 (30.7)	
Depression	Yes	317 (9.0)	85 (12.8)	9.52 (0.005)
	No	3102 (91.0)	620 (87.2)	
Suicidal thoughts	Yes	119 (2.9)	47 (6.5)	18.82 (<0.001)
	No	3300 (97.1)	658 (93.5)	
HRQoL		12.68 (0.07)	13.15 (0.14)	−2.99 (0.003)

**Table 3:** Factors affecting of HRQoL (*n* = 4131).

Characteristics		Daytime Work (*n* = 3425)		Shift Work (*n* = 706)	
		B	t (*p*)	B	t (*p*)
Age		−0.015	−0.96 (0.336)	0.002	0.05 (0.955)
Income level (lower)	Upper	0.002	0.01 (0.996)	0.775	1.04 (0.299)
	Middle	−0.552	−1.80 (0.073)	0.376	0.47 (0.636)
Education level (≥college)	≤Middle school	1.486	2.88 (0.005)	0.251	0.21 (0.834)
	High school	0.313	0.86 (0.387)	0.363	0.61 (0.537)
Living with spouse (no)	Yes	−1.253	−2.18 (0.030)	0.169	0.21 (0.831)
Intake of dietary supplements (no)	Yes	0.266	1.01 (0.313)	1.262	2.16 (0.032)
Occupation (technicians)	Manager	−0.082	−0.23 (0.817)	−0.505	−0.66 (0.508)
	Service workers	−0.583	−1.20 (0.230)	−0.796	−0.18 (0.237)
Regular workers (no)	Yes	−0.759	−2.43 (0.016)	−0.741	−1.20 (0.231)
Aerobic physical activity (yes)	No	−0.185	−0.67 (0.504)	0.644	1.06 (0.287)
Smoking (yes)	No	0.40	1.46 (0.146)	−0.689	−1.33 (0.183)
Depression (yes)	No	3.044	4.59 (<0.001)	0.833	0.41 (0.677)
Suicidal thoughts (yes)	No	2.921	3.76 (<0.001)	3.575	1.32 (0.188)
		R^2^ = 0.189, F = 7.29, *p* < 0.001		R^2^ = 0.176, F = 2.31, *p* = 0.007	

## Data Availability

The data presented in this study are available upon request from the corresponding author. The data are not publicly available because of privacy concerns.
